# “Growth From Failure”: A Qualitative Study of NICU Nurses’ Psychological Experiences Following Failed Neonatal Resuscitation

**DOI:** 10.1155/jonm/2056428

**Published:** 2026-10-29

**Authors:** Xushu Chen, Ru Yang, Zeyao Shi, Shulin Hou, Jing Yan, Xiaowen Li

**Affiliations:** ^1^ Department of Neonatology Nursing West China Second University Hospital Sichuan University Chengdu China scu.edu.cn; ^2^ Key Laboratory of Birth Defects and Related Diseases of Women and Children Sichuan University Ministry of Education Chengdu 610041 China meb.gov.tr

**Keywords:** NICU, nurses, psychological experience, qualitative research, resuscitation failure

## Abstract

**Aim:**

This study investigated the emotional responses and underlying psychological processes experienced by nurses in the neonatal intensive care unit (NICU) following unsuccessful neonatal resuscitation.

**Background:**

Nurses, particularly neonatal nurses, experience substantial psychological pressure and multifaceted challenges in the aftermath of resuscitation failure. Although existing literature has examined nurses’ emotional responses to neonatal death, a significant gap persists in understanding the distinct psychological experiences of neonatal nurses following unsuccessful resuscitation attempts.

**Methods:**

This was a descriptive phenomenological study. Purposive sampling was employed to recruit 14 NICU nurses from a tertiary hospital in Chengdu, Sichuan Province. Participants were selected based on their firsthand experiences of neonatal resuscitation failure and participated in semistructured, face‐to‐face interviews. The interviews were audio‐recorded, transcribed verbatim, and analyzed using Colaizzi’s seven‐step method.

**Results:**

NICU nurses described their personal experiences following failed neonatal resuscitation. Four themes emerged concerning nurses’ experiences and growth following resuscitation failure: (1) physical and mental exhaustion; (2) communication challenges; (3) reflection and support needs; and (4) achievements and growth.

**Conclusion:**

This study revealed the complex psychological experiences of NICU nurses following resuscitation failure. These findings highlight opportunities to support nurses’ professional growth and improve team culture and care systems. Consequently, establishing a multidimensional support system is important to support nurses involved in neonatal resuscitation.

**Implications for Nursing Management:**

Nurse managers and hospital administrators should establish a comprehensive, multidimensional support system to improve nursing competencies. This system should prioritize standardized training in the theoretical knowledge and practical skills needed for emergency care, while also strengthening nurses’ capacity to provide humanistic care.

## 1. Introduction

The World Health Organization (WHO) Global Report on Neonatal Health [1] indicates that approximately 15%–20% of newborns undergo resuscitation at least once within the first 28 days of life. The resuscitation rates for preterm and low‐birth‐weight infants were significantly higher than those for term infants [2]. In China, the incidence of neonatal resuscitation is 19.3%, with the majority of resuscitations occurring in the central and western regions of the country and in primary care settings [3]. The average resuscitation success rate in low‐ and middle‐income countries is approximately 50%–70%, owing to limitations such as insufficient equipment and inadequate training [4]. This means that at least 30% of the infants who undergo resuscitation do not survive [5].

Newborns often exhibit highly unstable vital signs due to the immaturity of their organ systems [6]. Guidelines indicate that common resuscitation measures include oxygen administration, electrocardiographic (ECG) monitoring, endotracheal intubation, chest compressions, and epinephrine administration [7]. Throughout this process, neonatal intensive care unit (NICU) nurses must collaborate closely and precisely with physicians. Under high‐pressure conditions, all critical procedures must be accurately performed within seconds. However, even with the implementation of advanced life support measures, including extracorporeal membrane oxygenation (ECMO), not all newborns can be successfully resuscitated [8]. This is especially true for extremely preterm infants with very low birth weight, for whom the challenges of resuscitation are substantially greater.

Nurses experience tremendous stress during resuscitation [9]. Following neonatal death, NICU nurses face significant mental health risks. Research indicates that up to 68% of nurses experience intense feelings of guilt and 52% report symptoms of anxiety [10]. During this period, nurses are typically in a state of high psychological vulnerability [11] because they are not adequately prepared to cope with such traumatic experiences [12]. However, nurses’ emotional burdens are frequently overlooked, and their support needs remain unmet. The cited report indicates a widespread lack of systematic support, with few dedicated mechanisms in place to help NICU nurses recover from these distressing situations [13]. Consequently, most nurses are left to process these intense negative emotions independently. The persistent accumulation of unaddressed negative emotions is likely to cause long‐term harm to nurses’ physical and mental well‐being and have a lasting adverse effect on their nursing practice [14].

The WHO notes that improving the quality of care is essential for enhancing newborn survival, health, and well‐being [15]. Therefore, it is necessary to focus on nurses’ cognitive and emotional responses to neonatal deaths [16]. Although phenomenological studies on neonatal death have gradually emerged, research on NICU nurses’ experiences of resuscitation failure and their emotional support needs remains insufficient. The death of a newborn profoundly affects the entire family unit. Consequently, the psychological experiences of NICU nurses following unsuccessful resuscitation are distinct and merit in‐depth exploration. This study employed semistructured interviews to explore the feelings and experiences of NICU nurses and to provide a theoretical basis for future research and support interventions.

## 2. Materials and Methods

### 2.1. Research Design

This qualitative study used a descriptive phenomenological approach. Descriptive phenomenology was deemed the optimal approach, as it enables researchers to capture the essence and meaning of shared lived experiences and aims to examine participants’ experiences from their perspective [17]. This approach is particularly well suited to gaining an in‐depth understanding of the unique experiences of NICU nurses, specifically their perceptions, feelings, and interpretations of unsuccessful neonatal resuscitation. This study was conducted from March 2025 to April 2025. Our study strictly followed the Consolidated Criteria for Reporting Qualitative Research (COREQ) checklist [18].

### 2.2. Setting and Sample

#### 2.2.1. Neonatal Resuscitation Team

Participants were recruited from three neonatal units at West China Second University Hospital in Chengdu, Sichuan Province, including the NICU and general neonatal wards. In our hospital, neonatal resuscitation is conducted by a dedicated team of at least five members, comprising a minimum of two physicians and three nurses. A team typically forms a U‐shaped configuration around the infant, with each member assuming a predefined role. The three nurses are responsible for (1) airway management (assisting physicians with procedures such as intubation), (2) medication administration (preparing and administering drugs), and (3) resuscitation assistant support (facilitating the transfer of supplies, securing the infant, and aiding in resuscitation efforts).

#### 2.2.2. Sample

Purposive sampling was used to recruit the participants. The inclusion criteria were as follows: (1) at least one year of clinical nursing experience in the NICU, (2) experience with at least one instance of neonatal resuscitation failure, and (3) the ability to fully express their feelings. The exclusion criteria were as follows: (1) current participation in an internship or further training, (2) being on sick or maternity leave, and (3) a history of mental illness. Gender, age, educational background, years of work experience, marital status, and job title were considered during participant selection.

### 2.3. Recruitment and Data Collection

#### 2.3.1. Recruitment

The participants were recruited through the following steps: First, an online questionnaire was distributed in the target wards to collect nurses’ general demographic information and experiences with neonatal resuscitation failure. The questionnaire was used for preliminarily screening of potential participants. Subsequently, the research team conducted face‐to‐face meetings with nurses who met the eligibility criteria. During these meetings, the study’s purpose, methodology, participant rights, and confidentiality principles were explained in detail, and informed consent was obtained. After the participants had been selected, individual appointments were made for one‐on‐one interviews. Because there is no fixed standard for sample size in qualitative research, we determined the sample size based on the principle of data saturation [19], which holds that sample size depends on the amount of information obtained.

#### 2.3.2. Data Collection

Individual, formal face‐to‐face interviews were conducted with all participants in the hospital conference room to facilitate open expression. Interviews were conducted by a trained qualitative researcher (Y.R.) between February 2025 and April 2025. The first author conducted all interviews to ensure consistency and accuracy [20, 21]. With the participants’ consent, the interviews were audio‐recorded. We used an interview guide to guide the main questions and flexibly adjusted the questions based on the interviewees’ responses. We consistently avoided using leading or suggestive questions and encouraged the participants to express their genuine feelings. When a discussion strayed from the topic, the researcher tactfully steered the conversation back on track to ensure that the interviews remained productive and focused on the research objective. Each interview lasted between 30 and 60 min, and data collection and analysis were conducted concurrently. The researchers also took observational notes on the participants’ facial expressions and body language during the interviews. No repeat interviews were conducted. The interviews were transcribed, and the transcripts were returned to the participants for accuracy verification.

#### 2.3.3. Interview Guide

Data collection followed a flexible interview guide. The interview guide was based on previous literature and was revised in consultation with neonatologists and nurses. Pilot interviews were conducted with two participants who were excluded from the final sample to assess the applicability of the interview guide. The guide included the following questions: (1) Have you experienced failed neonatal resuscitation? How many times have you experienced this? (2) What were the circumstances? Can you tell us about your personal experiences with failed resuscitation? (3) How did you feel after experiencing resuscitation failure? (4) What did you do after the infant in your care died? How did you feel when you met the infants’ parents? and (5) How did this experience change or affect your work and life? The interviews were conducted in Mandarin or the Sichuan dialect, depending on the participants’ preferences. To protect participants’ confidentiality, they were identified using the codes N1–N14.

### 2.4. Data Management and Storage

The researcher double‐checked the transcripts for accuracy. Unique study codes were assigned to each participant. All identifiable personal information was removed, and content that could reveal participants’ identities was obscured. The transcripts are password‐protected and can only be accessed by authorized data coders.

### 2.5. The Research Team

The research team comprised six members: Three held master’s degrees, and three were neonatal nurses with 6, 7, and 20 years of clinical experience in neonatology. Members with master’s degrees provided methodological leadership in study design, data collection, and analysis. The entire team collaboratively developed the interview guide and participated in interpretive validation.

### 2.6. Data Analysis

Within 24 h of each interview, two researchers (C.X.S. and Y.R.) independently reviewed the audio recordings, which were then imported into NVivo 12 (QSR International) for systematic management and analysis. The data analysis followed Colaizzi’s seven‐step phenomenological method [22]. The researchers thoroughly familiarized themselves with the data provided by the NICU nurses through repeated reading (Step 1). They analyzed the data sentence by sentence to identify and extract meaningful statements closely related to the research question (Step 2). Subsequently, recurring key themes were coded as preliminary units of meaning (Step 3). Similar codes were clustered into themes, theme groups, and categories to establish logical connections (Step 4). The intrinsic relationships between the themes and participants were described in detail to ensure a comprehensive interpretation. Subsequently, the interrelationships among the elements were analyzed in depth (Step 5), and similar themes and their descriptions were repeatedly compared to identify and construct concise phrases that captured their essential meaning (Step 6). Finally, the research themes were presented to the study participants to verify whether they accurately captured their experiences (Step 7).

### 2.7. Data Trustworthiness and Rigor

Four key criteria were used to ensure the rigor of this study [23, 24]. Credibility: The interview guide was developed through a literature review and reviewed by a panel of experts (*n* = 4) to ensure that the questions were appropriately designed. All participants underwent member checking, in which they reviewed thematic summaries and selected quotations to verify that our interpretations aligned with their experiences. Participants’ feedback was incorporated into the final analysis. For example, based on the participants’ suggestions, we revised the wording of the theme “Physical and mental exhaustion” to reflect their experiences more accurately. Confirmability was supported by independent data analysis by two researchers and team consensus on the final results. In addition, each researcher explicitly documented their preconceptions regarding resuscitation experiences prior to the analysis and reflected on how these assumptions might influence interpretations throughout the research process. For example, one researcher had previously worked as a neonatal nurse, and the team paid particular attention to the potential interpretive biases associated with this experience. Reliability: We supported our interpretations by directly quoting the participants’ original statements to ensure that our interpretations were grounded in the data. When interpretations differed, a consensus was reached by referring to the raw data and engaging in group discussions. Generalizability: We provided a detailed description of the research context, participant selection criteria, demographic characteristics, and the entire process of data collection and analysis. These measures enhanced the objectivity and accuracy of the research [25].

### 2.8. Ethics

The study adhered to the principles of informed consent. Before each interview, written informed consent was obtained from all participants, who were explicitly informed of their right to withdraw at any time without penalty. Given the sensitive nature of this research topic, a strict participant protection protocol was implemented. Throughout the interviews, the researcher closely monitored the participants for any signs of distress and promptly paused the session if a participant experienced any discomfort. Ethical approval was obtained from the Scientific Ethics Committee of West China Second Hospital of Sichuan University (YXKY: 2025044).

## 3. Results

The demographic characteristics of NICU nurses and related information are presented in Table 1. A total of 14 nurses (11 women and three men) were interviewed in this study, with ages ranging from 23 to 37 years, with a mean age of 30.29 ± 3.85 years, and were assigned numbers N1–N14.

**TABLE 1:** Demographic characteristics and relevant information on the interviewees.

No.	Gender	Age	Education level	Work year	Title	Marital status	Children
N1	Female	29	Bachelor’s degree	9	Senior nurse	Unmarried	Yes
N2	Female	23	Junior college	2	Junior nurse	Unmarried	No
N3	Female	33	Bachelor’s degree	12	Nurse in charge	Married	Yes
N4	Male	31	Bachelor’s degree	8	Nurse in charge	Married	No
N5	Female	29	Junior college	5	Senior nurse	Married	Yes
N6	Female	34	Bachelor’s degree	7	Senior nurse	Married	Yes
N7	Female	37	Bachelor’s degree	15	Senior nurse	Unmarried	No
N8	Female	29	Bachelor’s degree	5	Senior nurse	Unmarried	No
N9	Female	28	Bachelor’s degree	5	Senior nurse	Married	No
N10	Female	29	Bachelor’s degree	6	Senior nurse	Married	Yes
N11	Female	30	Bachelor’s degree	6	Nurse in charge	Married	Yes
N12	Female	37	Bachelor’s degree	17	Nurse in charge	Married	Yes
N13	Male	27	Bachelor’s degree	5	Senior nurse	Unmarried	No
N14	Male	28	Bachelor’s degree	5	Senior nurse	Married	Yes

Based on interviews with 14 participants, four themes and 11 subthemes emerged describing the psychological experiences of NICU nurses following neonatal resuscitation failure. These themes reflected the dynamic psychological experiences of nurses throughout this period: (1) physical and mental exhaustion; (2) communication challenges; (3) reflection and support needs; and (4) achievements and growth (see Figure 1).

**FIGURE 1:**
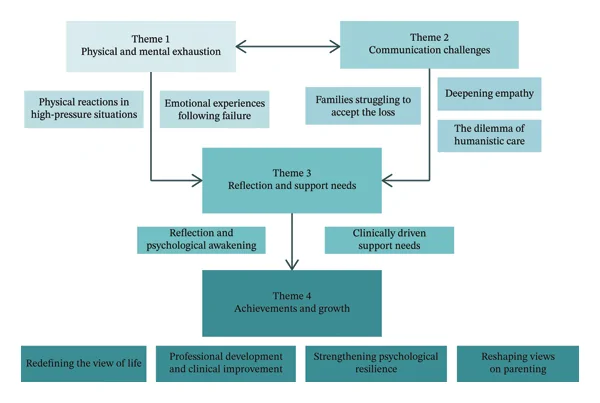
Thematic map of the psychological experiences of NICU nurses following neonatal resuscitation failure.

### 3.1. Theme 1: Physical and Mental Exhaustion

This study provides a detailed account of the physical and emotional exhaustion experienced by NICU nurses after failed resuscitation attempts. This theme encompasses both physical responses to high‐stress environments and emotional experiences following such failures.

#### 3.1.1. Subtheme 1: Physical Reactions in High‐Pressure Situations

Neonatal resuscitation is more challenging than adult resuscitation because the condition of a newborn can change rapidly and these changes are often difficult to detect. N2 described the sudden onset of the emergency: “*I am too busy with the emergency to have time to think.*” (N2). There were differences between the actual resuscitation process and the training. “*It is pretty chaotic at the scene, and (other) doctors have arrived as well.*” (N6). N8 added that resuscitation procedures are typically lengthy and grueling. “D*uring the resuscitation efforts, every second felt like an eternity because the resuscitation just kept going on and on…*” (N8). Nurses described involuntary physical reactions in high‐pressure situations. “*My hands just keep shaking and shaking—I am so nervous…*” (N4). Nurses also described physical exhaustion following intensive resuscitation. “*Actually, the whole (rescue) process was pretty exhausting.*” (N13). This high‐intensity resuscitation work provides valuable experience for future nursing practice.

#### 3.1.2. Subtheme 2: Emotional Experiences Following Failure

Although some resuscitation attempts are successful, others may fail even when the entire team has exhausted all possible measures. Most nurses who had experienced failed resuscitation reported varying degrees of emotional distress and psychological impact. “*But there really aren’t any other ways to save him.*” (N5). Grief was a prominent emotional response. “*Because at the time, I was crying while trying to save him.*” (N1). In addition, witnessing a newborn’s death firsthand often left a lasting impression on the nurses. “*The experience of being there in person is completely different from* what *you read in books. Witnessing a child’s death firsthand leaves a deep and lasting impression.*” (N1). One nurse described the visual appearance of the infant. “*It was covered in mottled patches—pale gray and slightly swollen.*” (N4). These profound and complex emotional experiences affected the nurses’ mental well‐being to varying degrees.

### 3.2. Theme 2: Communication Challenges

This theme captured the experiences of NICU nurses as they communicated with infants’ families following failed resuscitation attempts. These included families struggling to accept the loss, deepening empathy, shifting professional perspectives, and the dilemma of humanistic care. Physical and mental exhaustion compounded with communication challenges, creating a double burden.

#### 3.2.1. Subtheme 1: Families Struggling to Accept the Loss

Faced with sudden, devastating news, families often did not know how to cope. “*Most of them are first-time parents, and he (the family member) has no idea what to do.*” (N2). Although some parents had prepared themselves emotionally to some extent, they still find it difficult to accept the loss when it occurred. “*Some family members may have had a strong emotional reaction at the time and simply couldn’t accept it.*” (N14). This psychological disparity constitutes a core dilemma that nurses must confront during communication with bereaved families.

#### 3.2.2. Subtheme 2: Deepening Empathy

Nurses are not cold machines but warm and emotional human beings. “*When I see family members crying, I can’t help but cry too.*” (N7). Some nurses, who were mothers themselves, compared the sick infants with their own children, which heightened their empathy. “*It makes me think of my own child. It makes me think: Thank goodness I have a healthy child.*” (N3). This shift in nurse’s professional perspectives further reflects their deepening empathy.

#### 3.2.3. Subtheme 3: The Dilemma of Humanistic Care

Providing warmth and support to families during an infant’s final moments embodies the value of humanistic care. However, some nurses did not know how to communicate with infants’ families. “*I didn’t know how to comfort him either. To be honest, I’m not very good at comforting people.*” (N9). Some also felt awkward. “*Actually, sometimes we find ourselves in awkward situations given the circumstances at the time—no family member would accept such an outcome… The truth is, we rarely communicate with the families.*” (N4). However, other nurses chose to express their care nonverbally through gestures, companionship, or attentive care. “*Just get him a stool, help him up, and have him sit there (next to the baby incubator). Actually, everyone understood that at the time.*” (N4). These accounts suggest a gap between recognizing the importance of humanistic care and knowing how to provide it in practice.

### 3.3. Theme 3: Reflection and Support Needs

This theme described the debriefing experiences of NICU nurses following resuscitation attempts. This phase marked a turning point—from passively accepting the status quo to actively seeking change.

#### 3.3.1. Subtheme 1: Reflection and Psychological Awakening

Acknowledging one’s shortcomings can support professional growth. Almost all participants stated that they reflected on their performance after the event. “*I’ll reflect on it. I’ll think about what I could have done a little faster and where I could have done better.*” (N3). One nurse described feeling out of practice after returning from maternity leave: “*Since I had just returned from maternity leave and might have had to perform emergency resuscitation, I felt a little out of practice, so I was really nervous at the time.*” (N10). Another nurse noted that families’ emotional support needs could be overlooked. “*However, since it was my first time performing emergency resuscitation and I lacked experience, I didn’t communicate with the family or provide them with emotional support or care.*” (N1). This self‐reflection may help nurses identify areas for improvement in future resuscitation efforts.

#### 3.3.2. Subtheme 2: Clinically Driven Support Needs

Successful resuscitation requires support from multiple parties. Participants expressed a need for research and training that better supported clinical practice: “*We would prefer that both our research and our training be more closely aligned with clinical practice and provide more practical guidance for clinical work.*” (N12). In addition, participants emphasized the need to provide compassionate care and maintain the dignity of pediatric patients. “*Our department hasn’t handled the deaths of pediatric patients very well. After all, they aren’t just cats or dogs. They are living beings.*” (N6).

### 3.4. Theme 4: Achievements and Growth

Reflection and support can facilitate this transformation. When nurses receive adequate support and engage in ongoing reflection, they can achieve personal and professional growth after trauma. This theme describes how nurses perceived their experiences of failure as contributing to personal growth, laying the foundation for the overarching theme of “Growing Through Failure.”

#### 3.4.1. Subtheme 1: Redefining Perspectives on Life

Nurses described gaining new insights into life following failed resuscitation. Recognizing newborns’ potential alongside their vulnerability deepened their compassion. “*It’s just that you feel a newborn has limitless potential, but at the same time, you also feel they’re very fragile. It’s two extremes.*” (N8). Some nurses viewed death following unsuccessful resuscitation as an end to the infant’s suffering. “*I just feel that the child is at peace now, and he no longer has to suffer from his illness*.” (N9). This profound realization represents a soul‐searching inquiry into the limits of human strength and the essence of life itself.

#### 3.4.2. Subtheme 2: Professional Development and Clinical Improvement

Nurses described developing greater psychological resilience and becoming less fearful of resuscitation attempts following experiences of failure. “*After that, I became a little more resilient, and I wasn’t afraid to face emergency treatment again later on.*” (N4). Nurses also learned the importance of managing their emotional responses while maintaining empathy. “*In our line of work, you can’t be too empathetic, because it can affect our work and our mood.*” (N1). Moreover, new ideas emerged regarding improvements in clinical practice. “*I had some new ideas regarding the operations or processes. That’s probably why we ended up changing so many processes. I just felt that, at the time, I was actually quite eager to get that version out.*” (N12). These ideas informed changes to clinical processes. Nurses continuously refined their skills and grew through these failures.

#### 3.4.3. Subtheme 3: Strengthening Psychological Resilience

After experiencing trauma due to failed resuscitation, some nurses developed greater psychological adaptability. “*(I) take things in stride, and my outlook improves a bit.*” (N11). They also learned to consider the outcomes of resuscitation in the context of prognosis and its implications for families. “*Even if the resuscitation is successful, what difference does it make for the child—given the poor prognosis? And what about the family—the burden? Considering the financial strain on the family, the child’s prognosis, and the additional burden on society.*” (N4). However, reliance on self‐regulation may also indicate a gap in formal support for nurses coping with occupational trauma.

#### 3.4.4. Subtheme 4: Reshaping Views on Parenting

NICU nurses’ views on parenting were shaped by their professional experiences. Nurses who already had children described prioritizing their children’s health over other expectations. “*When it comes to my own kid, I just* want *him to be healthy—that’s all that matters. Nothing else really matters.*” (N11). They also emphasized valuing their children’s health and happiness over other achievements. “*Especially for those of us who work at hospitals—and particularly those of us with children. We set the bar very low for our own kids. We just think that as long as they’re healthy and happy, that’s enough. Everything else is just a bonus.*” (N6).

## 4. Discussion

To our knowledge, this is the first study to investigate the complex psychological experiences of NICU nurses following neonatal deaths after unsuccessful resuscitation. This study was informed by posttraumatic growth theory [26]. Our study identified four themes that illuminate a dynamic journey after failed resuscitation. Participants’ accounts described a progression from physical and emotional depletion during high‐pressure resuscitation through communication difficulties with families to reflective awareness and a need for support, ultimately arriving at a sense of achievement and professional growth. Together, these four themes outlined the nurses’ psychological journey “from failure to growth” following a failed resuscitation attempt. Our results provide practical guidance for healthcare institutions and nurse managers to develop coordinated support systems and psychological intervention strategies.

This study found that NICU nurses experienced physical and physiological distress during failed resuscitation events, consistent with the findings of Karlsson et al. [27] Intense mental strain can make nurses feel that time passes slowly during resuscitation efforts. At the same time, the hectic pace of emergency procedures takes a physical toll, causing symptoms such as muscle strain from prolonged cardiopulmonary resuscitation (CPR) and disruption of circadian rhythms [28]. In addition, the precise procedural requirements specific to neonatal resuscitation further amplify the intensity of stress [29]. Resuscitation efforts also impose immense pressure, which can trigger tension, fear, and shock among nurses. These reactions are frequently associated with limited experience in emergencies and feelings of being underprepared [28].

Notably, effective communication with families is often overlooked [30]. Our interviews indicated that novice nurses may have difficulty communicating with families and providing emotional support [31]. Therefore, nurses should thoroughly assess the values and preferences of the infant’s family so that they can provide personalized end‐of‐life care tailored to the specific circumstances of both the infant and the family [32]. Additionally, some studies have indicated that nurse managers can provide end‐of‐life care education to nurses [33, 34]. This education can be provided formally through nursing curricula or informally in clinical practice [35].

Our research findings suggest that reflection and support seeking can contribute to nurses’ growth after a failed resuscitation attempt. Conservation of resources theory [36] suggests that when individuals experience a significant depletion of resources, such as physical energy, emotional well‐being, skills, and social support, they tend to seek external resources to replenish and restore them. Consequently, when nurses are unable to cope on their own, this imbalance in resources may prompt them to reconstruct meaning through reflection and seek support to replenish their resources, potentially alleviating physical and mental exhaustion and helping them overcome communication barriers. However, obtaining support from nursing managers and organizations remains a major challenge in the context of resuscitation [37].

Despite the unsuccessful resuscitation attempts, nurses described learning and growth through reflection on their experiences. This is also consistent with the Chinese proverb “failure is the mother of success.” These findings are also consistent with Benner’s theory of nursing competence development, “From Novice to Expert.” [38] Our interviews indicated that reflection may help strengthen nurses’ psychological resilience and better prepare them for future resuscitation efforts [39].

## 5. Limitations

This study has some limitations. First, the sample was drawn from a single tertiary hospital, which may limit the generalizability of our findings. Second, the research focused exclusively on nurses. Future studies could adopt a mixed‐methods approach to examine changes in nurses’ psychological adjustment over time and incorporate parental perspectives to enhance the quality and compassionate delivery of care. Third, a history of mental illness was assessed solely through self‐report, which may have affected the consistency of eligibility assessment. In future research, with appropriate ethical approval and informed consent, screening could be strengthened using brief standardized tools such as the GHQ‐12 or PHQ‐9.

## 6. Conclusion

This study highlights the need to build a comprehensive support system incorporating institutional support, skills training, and end‐of‐life care education for NICU nurses following unsuccessful resuscitation. Participants’ accounts suggest that their psychological experiences may involve a nonlinear progression from failure to growth. Such support may help nurses provide compassionate end‐of‐life care and support bereaved families.

## 7. Implications for Nursing Management

Nurse managers should respond constructively to unsuccessful resuscitation by supporting nurses’ learning and professional growth. Based on the results of our study, we recommend that nurse managers provide NICU nurses with emergency care education and training in communication with families [40]. Psychological support should also be provided [41]. Ultimately, the goal is to help nurses learn from these experiences and continue their professional development [42].

## Author Contributions

Xushu Chen: writing–original draft, writing–review and editing, data curation, and formal analysis. Ru Yang: project administration, data curation, and investigation. Zeyao Shi: resources, supervision, and writing–review and editing. Shulin Hou and Jing Yan: writing–review and editing and investigation. Xiaowen Li: data curation, writing–review and editing, resources, and supervision.

## Funding

The authors have nothing to report.

## Ethics Statement

This study was approved by the Medical Research Ethics Committee of West China Second Hospital of Sichuan University (YXKY: 2025044). All participants provided informed consent before participating in the interviews. All study procedures were conducted in accordance with the relevant guidelines and regulations.

## Conflicts of Interest

The authors declare no conflicts of interest.

## Data Availability

Data are available from the authors upon request.

## References

[1] WHO , Improving Maternal and Newborn Health and Survival and Reducing Stillbirth–Progress Report 2023, 2023, WHO.

[2] Kamath-Rayne B. D. , Berkelhamer S. K. , Kc A. , Ersdal H. L. , and Niermeyer S. , Neonatal Resuscitation in Global Health Settings: an Examination of the Past to Prepare for the Future, Pediatric Research. (2017) 82, no. 2, 194–200, 10.1038/pr.2017.48. 28419084

[3] Wang Y. W. , Chen Y. , Ming Y. H. et al., Epidemiology and Region-Specific Risk Factors for Low Apgar Scores in China: A Nationwide Study, World Journal of Pediatrics. (2022) 18, no. 2, 135–141, 10.1007/s12519-021-00497-y. 34985717

[4] Kamath-Rayne B. D. , Thukral A. , Visick M. K. et al., Helping Babies Breathe, Second Edition: A Model for Strengthening Educational Programs to Increase Global Newborn Survival, Global Health: Science and Practice. (2018) 6, no. 3, 538–551, 10.9745/ghsp-d-18-00147. PMC6172134 30287531

[5] Zhang J. , Cao Y. , Su M. , Cheng J. , and Yao N. , The Experiences of Clinical Nurses Coping With Patient Death in the Context of Rising Hospital Deaths in China: A Qualitative Study, BMC Palliative Care. (2022) 21, no. 1, 10.1186/s12904-022-01054-8. PMC9494800 36138401

[6] Juvé-Udina M. E. , Fabrellas-Padrés N. , Delgado-Hito P. et al., Newborn Physiological Immaturity: A Concept Analysis, Advances in Neonatal Care. (2015) 15, no. 2, 86–93, 10.1097/anc.0000000000000162. PMC4961275 25822514

[7] Lee H. C. , Strand M. L. , Finan E. et al., Part 5: Neonatal Resuscitation: 2025 American Heart Association and American Academy of Pediatrics Guidelines for Cardiopulmonary Resuscitation and Emergency Cardiovascular Care, Circulation. (2025) 152, no. 16_suppl_2, S385–S423, 10.1161/cir.0000000000001367. 41122887

[8] Diaz-Miron J. , Reedy J. , Bennet T. D. , and DeCamp L. R. , When I Walk Away and Wonder, did I Make the Best Decision?: A Decisional Needs Assessment Among Providers Involved in Pediatric Extracorporeal Cardiopulmonary Resuscitation, Journal of Pediatric Surgery. (2025) 61, no. 1, 10.1016/j.jpedsurg.2025.162525. PMC12483763 40812407

[9] El-Ashry A. M. , Elsayed S. M. , Ghoneam M. A. , and Atta M. , Compassion Fatigue and Stress Related to Cardiopulmonary Resuscitation: A Study of Critical Care Nurses’ Experiences, BMC Nursing. (2023) 22, no. 1, 10.1186/s12912-023-01640-y. PMC10726549 38110907

[10] Wilson J. and Kirshbaum M. , Effects of Patient Death on Nursing Staff: A Literature Review, British Journal of Nursing. (2011) 20, no. 9, 559–563, 10.12968/bjon.2011.20.9.559. 21647017

[11] Kaya M. D. , Çalışkan B. B. , Çekiç Y. , Küçük ÖG. , and Bağ B. , The Imprints of Neonatal Death: A Qualitative Study on Neonatal Intensive Care Nurses’ Experiences of Neonatal Death, Advances in Neonatal Care. (2025) 25, no. 6, 565–573. 10.1097/ANC.0000000000001310 41066773

[12] Köktürk D. B. , Can Ş. , and Durgun H. , Emotional Responses of Neonatal Intensive Care Nurses to Neonatal Death, OMEGA–Journal of Death and Dying. (2022) 85, no. 2, 497–513. 10.1177/0030222820971880 33161878

[13] Yenal K. , Tektaş P. , Dönmez A. , and Okumuş H. , Perinatal Loss: Experiences of Midwives and Nurses, OMEGA–Journal of Death and Dying. (2023) 87, no. 4, 1174–1188, 10.1177/00302228211029143. 34324400

[14] Burke J. R. , Downey C. , and Almoudaris A. M. , Failure to Rescue Deteriorating Patients: A Systematic Review of Root Causes and Improvement Strategies, Journal of Patient Safety. (2022) 18, no. 1, e140–e155, 10.1097/pts.0000000000000720. 32453105

[15] Delafontaine A. C. , Anders R. , Mathieu B. , Salathé C. R. , and Putois B. , Impact of Confrontation to Patient Suffering and Death on Wellbeing and Burnout in Professionals: A Cross-Sectional Study, BMC Palliative Care. (2024) 23, no. 1, 10.1186/s12904-024-01393-8. PMC10941396 38486209

[16] WHO , Newborns: Improving Survival and Well-Being, 2025, WHO.

[17] Sundler A. J. , Lindberg E. , Nilsson C. , and Palmér L. , Qualitative Thematic Analysis Based on Descriptive Phenomenology, Nursing Open. (2019) 6, no. 3, 733–739, 10.1002/nop2.275. PMC6650661 31367394

[18] Tong A. , Sainsbury P. , and Craig J. , Consolidated Criteria for Reporting Qualitative Research (COREQ): A 32-Item Checklist for Interviews and Focus Groups, International Journal for Quality in Health Care. (2007) 19, no. 6, 349–357, 10.1093/intqhc/mzm042. 17872937

[19] Malterud K. , Siersma V. D. , and Guassora A. D. , Sample Size in Qualitative Interview Studies: Guided by Information Power, Qualitative Health Research. (2016) 26, no. 13, 1753–1760, 10.1177/1049732315617444. 26613970

[20] Fu M. R. and Rosedale M. , Breast Cancer Survivors’ Experiences of Lymphedema-Related Symptoms, Journal of Pain and Symptom Management. (2009) 38, no. 6, 849–859, 10.1016/j.jpainsymman.2009.04.030. PMC2795115 19819668

[21] Fu M. R. , Xu B. , Liu Y. , and Haber J. , Making the Best of it’: Chinese Women’s Experiences of Adjusting to Breast Cancer Diagnosis and Treatment, Journal of Advanced Nursing. (2008) 63, no. 2, 155–165, 10.1111/j.1365-2648.2008.04647.x. 18537844

[22] Colaizzi P. , Psychological Research as a Phenomenologist Views it. Existential Phenomenological Alternatives for Psychology, 1978.

[23] Lincoln Y. and Guba E. , But is it Rigorous? Trustworthiness and Authenticity in Naturalistic Evaluation, New Directions for Program Evaluation. (1986) 1986, no. 30, 73–84, 10.1002/ev.1427.

[24] Graneheim U. H. and Lundman B. , Qualitative Content Analysis in Nursing Research: Concepts, Procedures and Measures to Achieve Trustworthiness, Nurse Education Today. (2004) 24, no. 2, 105–112, 10.1016/j.nedt.2003.10.001. 14769454

[25] Barrett A. , Kajamaa A. , and Johnston J. , How to…be Reflexive When Conducting Qualitative Research, Clinical Teacher. (2020) 17, no. 1, 9–12, 10.1111/tct.13133. 31970937

[26] Tedeschi R. G. and Calhoun L. G. , The Posttraumatic Growth Inventory: Measuring the Positive Legacy of Trauma, Journal of Traumatic Stress. (1996) 9, no. 3, 455–471, 10.1002/jts.2490090305. 8827649

[27] Karlsson L. , Gustafsson U. , Thernström B. Y. , Wallström L. , and Broström A. , Neonatal Resuscitation: A Critical Incident Technique Study Exploring Pediatric Registered Nurses’ Experiences and Actions, Advances in Neonatal Care. (2023) 23, no. 3, 220–228. 10.1097/ANC.0000000000001063 PMC10205119 36905225

[28] Koželj A. , Šikić P. M. , Fijan S. , Strauss M. , Poštuvan V. , and Strnad M. , Exploring the Feelings of Nurses During Resuscitation-a Cross-Sectional Study, Healthcare-Basel. (2021) 10, no. 1. 10.3390/healthcare10010005 PMC8774964 35052169

[29] Hainstock L. M. and Raval G. R. , Neonatal Resuscitation, Pediatrics in Review. (2020) 41, no. 3, 155–158, 10.1542/pir.2018-0203. 32123029

[30] Lu S. , Quinn M. , and Vente T. , Hiding Hospice: Culturally Responsive Care at Pediatric End of Life (CS329), Journal of Pain and Symptom Management. (2023) 65, no. 5, 10.1016/j.jpainsymman.2023.02.091.

[31] Adams L. , Suresh G. K. , and Lahey T. , Ethical Issues in Pediatric Global Health, Pediatric Clinics of North America. (2016) 63, no. 1, 195–208, 10.1016/j.pcl.2015.09.002. 26613697

[32] Brødsgaard A. , Pedersen J. T. , Larsen P. , and Weis J. , Parents’ and Nurses’ Experiences of Partnership in Neonatal Intensive Care Units: A Qualitative Review and Meta-Synthesis, Journal of Clinical Nursing. (2019) 28, no. 17-18, 3117–3139, 10.1111/jocn.14920. 31112337

[33] Burnitt E. , Grealish L. A. , Crilly J. , May K. , and Ranse J. , Providing End of Life Care in the Emergency Department: A Hermeneutic Phenomenological Study, Australasian Emergency Care. (2024) 27, no. 3, 161–166, 10.1016/j.auec.2024.01.002. 38310030

[34] Aquino J. , Crilly J. , and Ranse K. , The End-of-Life Care Practices of Emergency Care Nurses and the Factors that Influence These Practices: An Integrative Review, International Emergency Nursing. (2022) 63, 10.1016/j.ienj.2022.101168. 35584560

[35] Ranse K. , Delaney L. , Ranse J. , Coyer F. , and Yates P. , End-of-Life Care Content in Postgraduate Critical Care Nursing Programs: Structured Telephone Interviews to Evaluate Content-Informing Practice, Australian Critical Care. (2020) 33, no. 2, 181–186, 10.1016/j.aucc.2019.04.004. 31182250

[36] Hobfoll S. E. , Conservation of Resources. A New Attempt at Conceptualizing Stress, American Psychologist. (1989) 44, no. 3, 513–524, 10.1037/0003-066x.44.3.513. 2648906

[37] Zali M. , Rahmani A. , Hassankhani H. et al., Critical Care Nurses’ Experiences of Caring Challenges During Post-Resuscitation Period: A Qualitative Content Analysis, BMC Nursing. (2024) 23, no. 1, 10.1186/s12912-024-01814-2. PMC10910715 38433187

[38] Lamichhane G. , Shaping Professional Nursing Practice Using Benner’s Novice to Expert Theory, British Journal of Nursing. (2025) 34, no. 22, 1133–1137, 10.12968/bjon.2024.0387. 41355560

[39] Thomas C. M. and Kellgren M. , Benner’s Novice to Expert Model: An Application for Simulation Facilitators, Nursing Science Quarterly. (2017) 30, no. 3, 227–234, 10.1177/0894318417708410. 28899267

[40] Nordby H. and Nøhr Ø. , The Ethics of Resuscitation: How do Paramedics Experience Ethical Dilemmas When Faced With Cancer Patients with Cardiac Arrest?, Prehospital and Disaster Medicine. (2012) 27, no. 1, 64–70, 10.1017/s1049023x1200026x. 22591932

[41] Liu L. , Li M. , Peng M. , Yan X. , and Luo S. , Factors Influencing Critical Care Nurses’ Communication Difficulties With Mechanically Ventilated Patients: Nurses’ Perspectives, Applied Nursing Research. (2026) 88, 10.1016/j.apnr.2026.152069. 41826031

[42] Wang L. , Li S. , Liu X. , Li R. , and Li R. , The Mediating Role of Resilience in the Relationship Between Meaning in Life and Attitude Toward Death Among ICU Nurses: A Cross-Sectional Study, Frontiers in Psychology. (2024) 15, 10.3389/fpsyg.2024.1414989. PMC11570999 39559693

